# RGB-based visual encoding of vibration data for gearbox fault diagnosis using U-Net segmentation model

**DOI:** 10.1371/journal.pone.0350838

**Published:** 2026-06-22

**Authors:** İrfan Kiliç, Gülşah Karaduman, Beyda Tasar, Orhan Yaman

**Affiliations:** 1 Department of Software Engineering, Engineering Faculty, Firat University, Elazig, Turkey; 2 Department of Computer Engineering, Engineering Faculty, Firat University, Elazig, Turkey; 3 Department of Mechatronics Engineering, Engineering Faculty, Firat University, Elazig, Turkey; 4 Department of Digital Forensics Engineering, Technology Faculty, Firat University, Elazig, Turkey; CINVESTAV IPN: Centro de Investigacion y de Estudios Avanzados del Instituto Politecnico Nacional, MEXICO

## Abstract

This study presents an innovative approach for diagnosing gearbox gear faults by enabling numerical vibration data analysis using image-based deep learning models. The Gearbox Fault Diagnosis Data set available on Kaggle was used to collect vibration signals from four different sensors (a1, a2, a3, a4). The maximum, minimum, and mean values of these signals were calculated and normalized within the [0–255] range and then mapped to the red, green, and blue (RGB) color channels, respectively. As a result, 500 images of 256 × 256 pixels were generated for each category. Then, these image representations were used to train a pre-trained U-Net deep learning model for segmentation, with only 10 training epochs. The model achieved a classification accuracy of 99.87% and an mean average precision (mAP) score of 99.74%. These high-performance metrics demonstrate that converting non-visual numerical data into RGB images and analyzing them using convolutional neural networks (CNNs) offers significant advantages over commonly used machine learning and text-based deep learning methods.To the best of our knowledge, this is the first study to classify numerical sensor data with such high accuracy by converting it into a visual format. The proposed method not only advances the field of gearbox fault detection and introduces a new paradigm for solving similar signal-based engineering problems in the literature.

## 1. Introduction

Gearboxes are among the basic components in many industrial and precision applications in the industrial sector. With the impact of Industry 4.0, condition monitoring and fault diagnosis (AT) of rotating machines have gained importance [1–3]. These systems play a critical role in motion and power transmission in modern industrial mechanisms [2]. Spur gearboxes [4,5], helical gearboxes [6], bevel gearboxes [3,7], and planetary gearboxes [8] are preferred in different rotating machine applications because of their flexibility. Gearboxes play a central function in automation processes by increasing the mechanical system efficiency.

Due to harsh operating conditions and continuous loading, gearbox failures can occur in gearboxes [9]. Rolling and sliding movements; the sliding direction is usually in the opposite direction [10,11]. Insufficient lubrication damages the gear surfaces by increasing contact, temperature increase and wear on the surfaces. Tensile stress, surface properties, and the presence of defects in the gear roots affect the occurrence of failures [12,13]. Failures are generally classified as lubricated or unlubricated. Lubricated failures include problems such as insufficient lubrication and pitting, whereas unlubricated failures are associated with breakages due to excessive loads [10,14]. These failures can increase the vibration and noise levels of the system, leading to serious damage and economic losses [15–17]. Therefore, the development of fault diagnostic technologies is of vital importance in ensuring the safe operation of rotating machines and reducing maintenance costs [18]. In recent years, condition monitoring and fault diagnosis of gearboxes have been an intensive research topic.

### 1.1. Related works

Fault detection in rotating machinery is usually based on the analysis of various types of data, such as vibration data, oil and bearing temperatures, torque, vibration, and current signals. Vibration data-based approaches are the most widely used among these methods because they carry fault traces in the signal.

Vibration measurements are usually performed to identify gearbox failures, and these measurements are performed by multiple sensors. Although broken gears cause force pulses in the vibration signal, the accurate evaluation of these signals is a complex process.

Vibration signals in a gearbox exhibit nonlinear and Gaussian characteristics due to factors such as friction, damping, nonlinear stiffness, sudden peaks occurring in localized fault regions and load variations between gears. Such faults spread the energy over different frequencies, making it difficult to analyze the signal. Furthermore, because each gearbox produces a unique vibration signal, there is a high risk that methods or settings that are suitable for one system may fail in another system. To overcome these challenges, researchers have developed various methods that combine vibration signals, acoustic data, and signal processing techniques with machine learning algorithms. These approaches have resulted in remarkable achievements in fault detection processes. A summary of the studies in this field is given in Table 1.

**Table 1:** Summary of related works.

Reference	Used Data	Method	Success Rate (%)
Durbhaka et al. [19]	Acoustic emission and accelerometer dataRenewable energy turbine data	Deep random forest combination techniqueOptimized LSTM	97,6887,5
Vrba et al. [20]	Gearbox fault data	Support Vector Machine (SVM), Normalized least mean squares (NMLS)	100
Yu et al. [21]	Gearbox fault data	Multiscale fusion global sparse network (MFGSNet)	97,06
Yao et al. [22]	Acoustic-based data	Multi-scale convolutional learning structure	93,3
Ye et al. [23]	Gear vibration signals	AKRNet	99,19
Chen et al. [24]	Gearbox fault data	Combination of EMD, LSTM and PSO	97,44
Zhao et al. [25]	Planetary gearbox fault data	DRN+DWWC method	99,6
Liu et al. [26]	Noise cancelled signals	1D CNN	99,34
Wang et al. [27]	Planetary gearbox fault data	Time-frequency representation and deep reinforcement learning	99,95
He [28]	Fault detection data	Deep belief network optimized by genetic algorithm	100
Li et al. [29]	Pitting failures	Augmented deep sparse autoencoder (ADSAE)	
Saufi et al. [30]	Limited data samples	Stacked sparse autoencoder (SSAE)	100
Sun et al. [31]	Planetary gearbox fault data	IPVMD and I-CNN	97,1
Heydarzadeh et al. [32]	Vibration, acoustic and torque signals	DWT and deep neural network	97,31
Chen et al. [33]	Industrial gearbox fault data	CNN based method	98,9
Raji et al. [34]	Planetary gearbox fault data	VMD and CNN	98,75
Steven R et al. [35]	Accurate signal representation	EMD	
Ha et al. [36]	Gearbox fault data	Health data map and deep field harmonization	100
Shi et al. [37]	Spatial and temporal feature extraction	BiConvLSTM	84,72
Ye et al. [38]	Noise filtering, comparison with CNN architecture	DMCNet	98,61
Zhang et al. [39]	Wind turbine gearboxes	HA-ResNet	98,76
Chen et al. [40]	Planetary gearbox fault data	DWT and CNN combination	99,3
Yang [41]	Gear crack rate classification	Particle filters	
Azamfar [42]	Motor current signature analysis	CNN	250rpm:98.83, 500 rpm:98.79, 1000 rpm:100, 1500 rpm:94.58
Feng et al. [43]	Rotary encoder signals	Layout spectrum analysis	
Yao et al. [44]	Vibration signals	DE-KELM	98,125
Zhang et al. [45]	Short frequency Fourier and mode decomposition	Not specified	
Zhang [46]	Multimode data	Cross-domain junction network	96,6

### 1.2. Motivation and contributions

In previous studies, vibration data were trained using text-based classical machine learning and deep learning models to classify healthy and broken vibrations. Therefore, fault diagnostic performance could not produce state-of-the-art results. We believe that it may be possible to obtain state-of-the-art results in problems where only two classes are determined. Therefore, a method can be developed from a different perspective. The proposed method converts text data into image data and uses pre-trained deep learning models for segmentation of images. The following main contributions have been achieved with this study;

1. The maximum, average, and minimum values of the vibration data of different sensors are used.
2. Matching the Red, Green and Blue channels of the image with the normalized maximum, average, and minimum vibration data in the range [0–255].
3. Creating 500 pieces of 256x256 images for both classes from the matched data.
4. Segmenting the images by training the pre-trained U-Net deep learning segmentation model for the first time.

## 2. Material and method

### 2.1. Dataset

With the help of SpectraQuest’s Gearbox Fault Diagnostics Simulator, broken and healthy gear data were collected at 10 different loads [0, 10, 20, 30, 40, 50, 60, 70, 80, 90] with four different vibration sensors (a1, a2, a3, a4). Healthy gear data file name starts with ‘h’ (e.g., h30hz0.csv). Broken gear data file name starts with ‘b’ (e.g., b30hz0.csv). Table 2 shows the amount of vibration data obtained from the sensors.

**Table 2:** Gearbox gear vibration data.

Sensor	Healthy	Broken	Total
a1	88.832	88.320	177.152
a2	88.832	88.320	177.152
a3	88.832	88.320	177.152
a4	88.832	88.320	177.152
**Total**	355.328	353.280	708.608

Healthy refers to the normal operating state of the gearbox under different loads. Broken means that the gearbox performance at different loads is degraded due to broken tooth failure.

**Fig 1:**
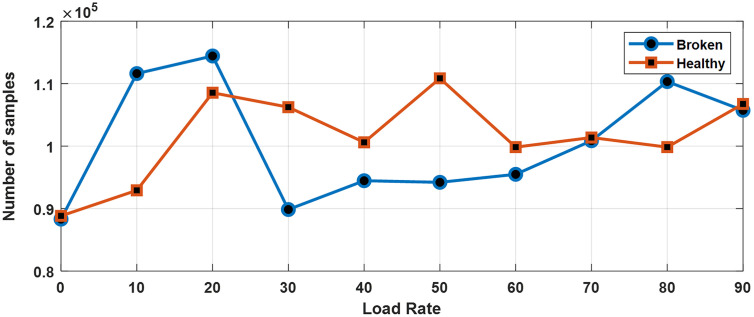
Number of healthy and defective gear data at different loads.

The data given in Table 2 were measured at different loads in the range [0–90] %. Fig 1 shows the data amount graph for different loads. The amount of data for different loads ranges from 90,000–115,000. In Fig 1, the horizontal axis represents the Load Rate increasing from 0 to 90, and the vertical axis represents the number of Broken and Healthy data points at these loads.

### 2.2. The Proposed method

**Fig 2:**
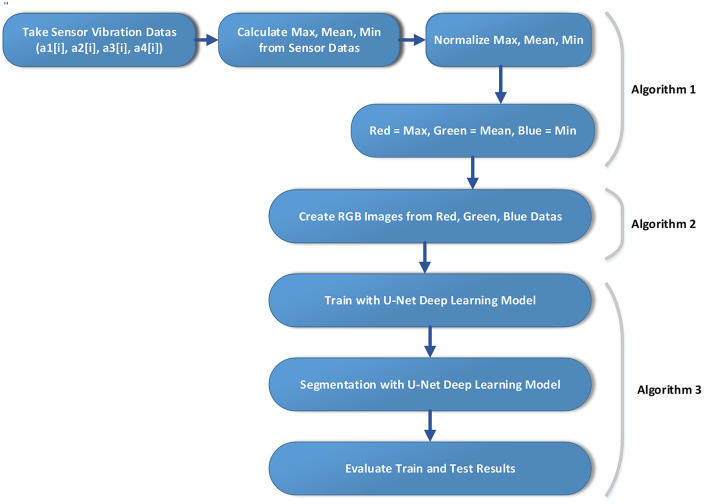
Proposed method framework.

In general, our proposed method can be expressed as converting non-visual sensor vibration data into RGB images and segmenting them by training with image-based deep learning models. Fig 2 shows the general framework of the proposed method.

Although the main problem is formulated as binary classification (healthy vs. faulty), the dataset inherently reflects a more complex scenario due to multiple load conditions, multi-sensor inputs, and large-scale variability. Therefore, binary classification was used as a controlled baseline for evaluating generalization, sensor fusion, and robustness under varying operating conditions. Instead of direct classification, a segmentation-based framework—where 1D vibration signals are converted into RGB images and learned through block-based spatial encoding—was employed as a representation learning strategy. This approach allows the model to capture local patterns, inter-sensor relationships, and distributional variations more effectively. Furthermore, the proposed methodology can be naturally extended to multi-failure scenarios by adapting the segmentation masks to multi-class structures. Compared to traditional 1D and time–frequency methods, the image-based representation provides a computationally efficient and structurally robust alternative for vibration-based fault diagnosis.

When Fig 2 is analyzed, 3 different algorithms were applied for our method. The first algorithm is the process for the transformation of vibration data into images (Algorithm 1). The pseudo-code of Algorithm 1 is given below.

**Algorithm 1:** Conversion of the sensor vibration data into R, G, and B datas

**Input:**      a1, a2, a3, and a4 sensor vibration data

**Output:**  Red, Green, Blue, Labels data

 1:   a1[], a2[], a3[], a[] = 0, sensor_data = LoadFile(“merged_csv_file”)

 2:      Red[], Green[], Blue[] = 0, Labels[]

 3:      MaxR, MeanG, MinB = 0

 4:      **for** i in length(sensor_data):

       a1[i] = sensor_data[i][1], a2[i] = sensor_data[i][2]

       a3[i] = sensor_data[i][3], a[i] = sensor_data[i][4]

       MaxR = max (a1[i], a2[i], a3[i], a4[i])

       MeanG = mean (a1[i], a2[i], a3[i], a4[i])

       MaxB = min (a1[i], a2[i], a3[i], a4[i])

       Labels[i] = sensor_data[i][5]

       Red[i] = Normalize (MaxR, [0–255])

       Green[i] = Normalize (MeanG, [0–255])

       Blue[i] = Normalize(MinB, [0–255])

 5:      **end for**

 6:      **return** Red[], Green[], Blue[], Labels[]

Algorithm 2 describes the creation of 500 images from the red, green, and blue data obtained. Algorithinvolvesbout training and segmenting the generated RGB images with the U-Net deep learning model. The data whose Max, Mean, and Min values are calculated are normalized to the range [0–255] since each channel is 8-bit before being equalized to the Red, Green, and Blue channels. While creating the image sizes, since the input of the U-Net deep learning model was 256x256, the images were created in 256x256 size. 80% of the generated images were used for training and 20% for testing. After the model training, segmented images in 4x4 dimensions were obtained.

Algorithm 2 was used to create labeled images with Red, Green, Blue and Labels data obtained according to Algorithm 1. The pseudo-code of Algorithm 2 is given below.

**Algorithm 2:** Conversion of Red, Green, Blue data to image

**Input:** Red[], Green[], Blue[], Labels[]

**Output:** 500 images with labels

 1:   Create folder “images” and “response”

 2:   *image_size = 256, num_images = 500, num_blocks = 4*

 3:   *block_size = image_size / num_blocks*

 4:   **for** *i* **in** *num_images*:

 4.1:    *red_channel[], green_channel[], blue_channel[] = 0, label_rgb[] = 0*

 4.2:    for j **in** *num_blocks x num_blocks*:

         calculate block start and end coordinates

         selected_class = Choose a random class (0 or 1)

         find indexes of this class

 4.2.1:     If there is enough data of this class **then**

           *bs =* randomly select ***block_size***^***2***^ of data (Red[], Green[], Blue[], Labels[])

           *red_channel[start..bs]* = Red[start..*bs*]

           *green_channel[start..bs]* = Green[start..block_*bs*]

           *blue_channel[start..bs]* = Blue[start..*bs*]

           label_rgb[start..bs] = selected_class*255

         End If

4.2.2:       *image[j]* = merge (*red_channel[], green_channel[], blue_channel[]*)

            response[j] = merge (*label_rgb[]*)

4.3:     end for

       *image_i* = merge *(image[j])*

       *response_i* = merge *(response[j])*

 4.4:  save_image(*image_i), save_image(response_i)*

 5:         end for

 6:         return images, labels

**Fig 3:**
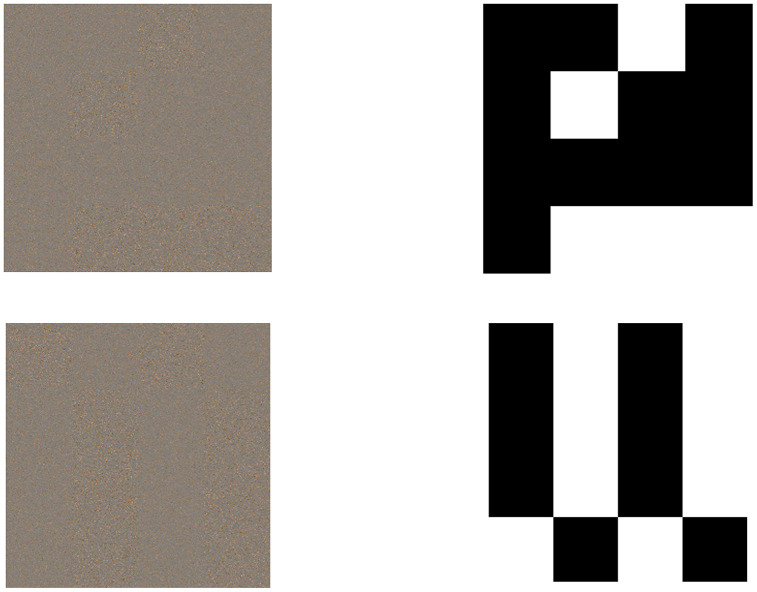
Generated images (a) Image (b) Ground Truth (Labels).

Sample images generated with Algorithm 2 are given in Fig 3. As shown in Fig 3, the generated images are Ground Truth images representing block-based class information. The black and white blocks represent the Broken and Healthy labels, respectively. The images and labels (classes) obtained according to Algorithm 2 were trained with the U-Net deep learning model according to Algorithm 3.

**Algorithm 3:** Training of images with U-Net

**Input:** images, responses

**Output:** metrics, labels

 1:   classNames = ["0", "1"]

          img_train = split (images, 1:400), img_test = split (images, 401:500)

          labels_train = split (responses, 1:400), labels_test = split (responses, 401:500)

 2:     training data = merge(img_train, labels_train)

 3:     numClasses = numel(classNames), imageSize = [256 256 3]

 4:     lgraph = unetLayers(imageSize, numClasses)

 5:   options = trainingOptions(’adam,’ ‘InitialLearnRate,’ 1e-4, ‘MaxEpochs,’ 10, ‘MiniBatchSize,’ 16, ‘Plots,’ ‘training-progress,’ ‘ValidationData,’ pixelLabelImageDatastore(img_test, labels_test), ‘ValidationFrequency,’ 10)

 6:   net = trainNetwork(trainingData, lgraph, options)

 7:   labels = semanticseg (img_test, net, ‘MiniBatchSize’, 16)

          metrics = evaluateSemanticSegmentation(labels, labels_test)

 7:     **return** metrics, labels

### 2.3. Full workflow description

**Fig 4:**
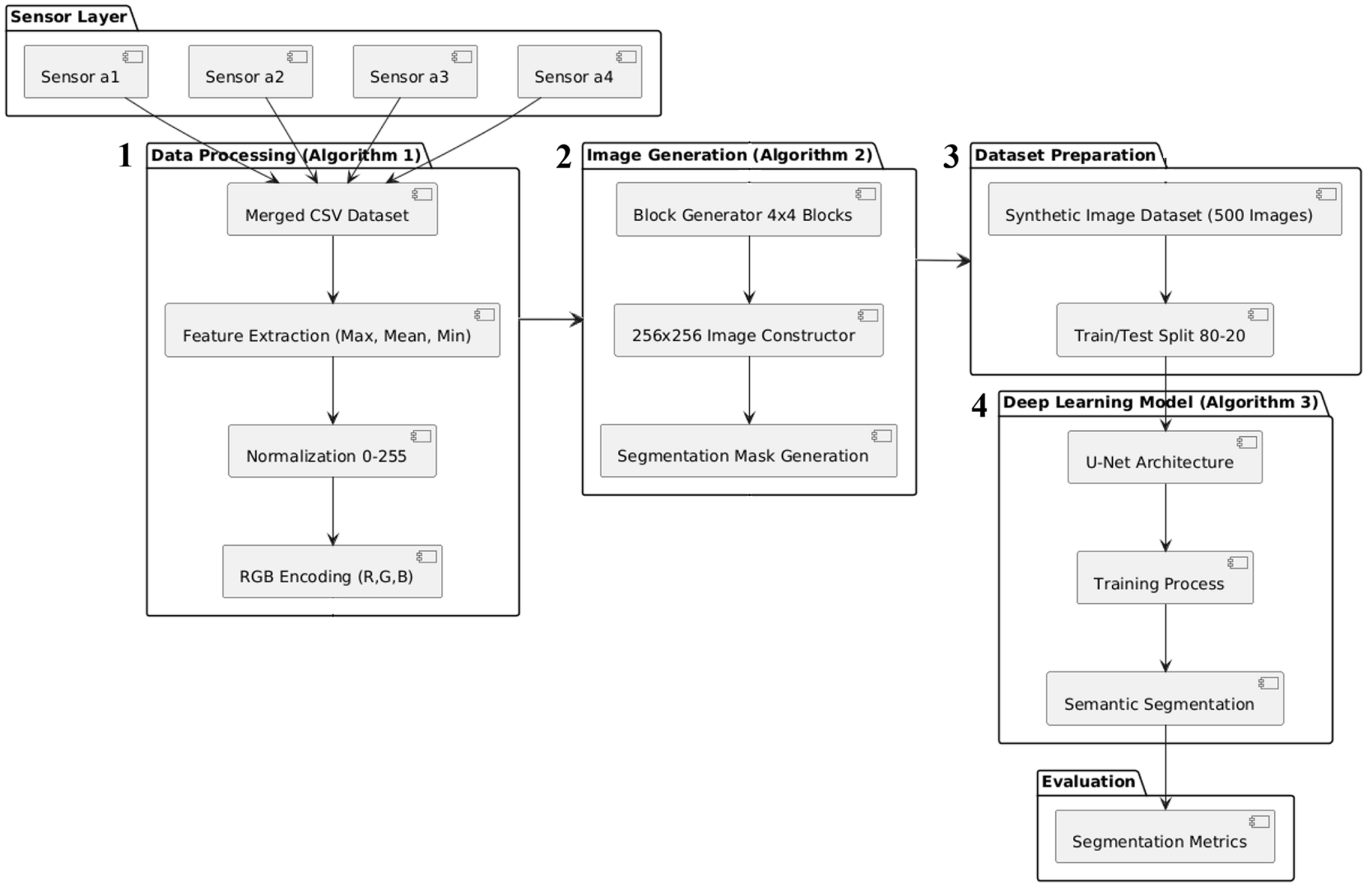
Workflow overview of the proposed method.

Fig 4 summarizes the general methodology we propose. As shown in Fig 4, the proposed method converts non-visual vibration sensor signals into RGB images and performs classification through semantic segmentation using a U-Net–based deep learning architecture. The complete workflow consists of four main stages: (i) data acquisition and preprocessing, (ii) feature extraction and RGB encoding, (iii) synthetic image generation, and (iv) deep learning–based segmentation and evaluation using performance metrics.

Combining raw data and preprocessing: The raw data consist of vibration signals collected from four sensors, denoted as ${\text{a}}_{\text{1}},{\text{a}}_{\text{2}},{\text{a}}_{\text{3}}$and ${\text{a}}_{\text{4}}$. The combined CSV file includes four synchronized vibration measurements along with class labels indicating fault conditions. After loading the combined dataset, the following preprocessing steps are applied, including parsing the sensor measurement data and label information (Algorithm 1):

- Load the combined vibration dataset
- Parse sensor data and labels
- Extract statistical features (max, mean, min)
- Normalize values to the RGB intensity range [0, 255]

No signal filtering is applied prior to feature extraction. The preprocessing pipeline can be summarized as follows:

Sensor vibration data → Feature extraction → Normalization → RGB channel mapping

For each index *i* (i.e., for each row), the four sensor values are used to calculate statistical features representing the current vibration state. Accordingly, the set can be defined as:


$$\begin{array}{c} {\text{V}}_{\text{i}}\;=\;\left\{{\text{a}}_{\text{1}}[\text{i}],{\text{a}}_{\text{2}}[\text{i}],{\text{a}}_{\text{3}}[\text{i}],{\text{a}}_{\text{4}}[\text{i}]\right\} \end{array}$$

The computation of the max, mean, and min statistical values is given in Equations (1)–(3).


$$\begin{array}{c} \text{Max}{\text{R}}_{\text{i}}\;=\text{ max}\left({\text{V}}_{\text{i}}\right) \end{array}$$ (1)

$$\begin{array}{c} \text{Mean}{\text{G}}_{\text{i}}\;=\;\frac{{\text{a}}_{\text{1}}[\text{i}]+{\text{a}}_{\text{2}}[\text{i}]+{\text{a}}_{\text{3}}[\text{i}]+{\text{a}}_{\text{4}}[\text{i}]}{\text{4}} \end{array}$$ (2)

$$\begin{array}{c} \text{Min}{\text{B}}_{\text{i}}\;=\text{ min}({\text{V}}_{\text{i}}) \end{array}$$ (3)

The obtained ${\text{R}}_{\text{i}}$, ${\text{G}}_{\text{i}}$, and ${\text{B}}_{\text{i}}$ values represent the vibration distribution at each time interval. These values are subsequently used for normalization and RGB encoding. An image consists of three channels R, G, and B each with an 8-bit depth. Therefore, the ${\text{R}}_{\text{i}}$, ${\text{G}}_{\text{i}}$, and ${\text{B}}_{\text{i}}$ values are normalized as defined in Equation (4).


$$\begin{array}{c} \text{I }=\text{ 255}\times \frac{\text{x}-{\text{x}}_{\text{min}}}{{\text{x}}_{\text{max}}-{\text{x}}_{\text{min}}} \end{array}$$ (4)

Here, *I* represents the image pixel, and *x* denotes the max, mean, and min values.

The normalized max, mean, and min values are then mapped to the R, G, and B channels, respectively. This mapping yields $\left({\text{R}}_{\text{i}},{\text{G}}_{\text{i}},{\text{B}}_{\text{i}}\right)$ pixel values for the *i*-th time interval. These pixels are used to construct images of size 256 × 256.

Each image is divided into 4 × 4 = 16 blocks, where each block contains 256/4 = 64 pixels along both the horizontal and vertical dimensions. Therefore, each image block has a size of 64 × 64 pixels.

In Algorithm 2, during block construction, each block is filled using vibration samples belonging to a selected class. The images created from these blocks are generated as follows:

1. Select a random class label (0 or 1)
2. Retrieve vibration samples corresponding to the selected class
3. Randomly select a sufficient number of RGB samples
4. Fill the corresponding block region with these RGB values
5. Assign the block class value in the segmentation mask

The segmentation mask classes are defined as:

- 0 → class 0 (broken)
- 255 → class 1 (healthy)

In this manner, each block corresponds to a uniform class region in the ground-truth mask.

Each vibration sample initially has a single class label. When samples are placed into image blocks:

- All pixels generated from these samples inherit the same class label
- The class label is assigned to the corresponding region in the segmentation mask

Thus, a fixed mask is generated on a block-wise basis. If a block corresponds to class 1, the mask pixel value is set to 255; for class 0, it is set to 0. In this way, vibration-based class labels are transformed into pixel-level segmentation masks.

**Rationale for Choosing Segmentation Instead of Direct Classification**: Although the problem involves two classes, a semantic segmentation framework is preferred over direct classification for the following reasons:

1. It enables learning of spatial distributions within synthetic images.
2. It improves robustness against noise in vibration patterns.
3. The U-Net architecture provides strong feature extraction through its encoder–decoder structure.
4. Block-based labeling allows the network to learn local vibration patterns rather than relying on a single global decision.

In total, 500 RGB images are generated. Of these, 80% (400 images) are used for training, and 20% (100 images) are used for testing. Since both image blocks and entire images are randomly generated during the image synthesis process, additional shuffling of the dataset is not required. The training images are used to train the U-Net model, while the test images are used for validation and evaluation.

The configuration of the U-Net network is as follows:

- Input size: 256 × 256 × 3
- Number of classes: 2
- Optimization algorithm: Adam
- Learning rate: 1 × 10 ⁻ ⁴
- Number of epochs: 10
- Mini-batch size: 16

Performance is evaluated using semantic segmentation metrics.

The overall workflow can be summarized as follows:

1. Load the combined vibration dataset
2. Separate sensor channels and labels
3. Compute maximum, mean, and minimum features
4. Normalize features to the [0–255] range
5. Map features to RGB channels
6. Generate 500 synthetic 256 × 256 RGB images (total of 16 blocks per image)
7. Create corresponding segmentation masks using block-based labeling
8. Split the dataset into training and test sets
9. Train the U-Net segmentation model
10. Perform segmentation on the test images
11. Evaluate performance using segmentation metrics

### 2.4. U-Net image segmentation deep learning model

U-Net is a convolutional neural network model that is especially used in medical image segmentation [47–50]. Segmentation of a 512 × 512 image can be performed on a graphics processing unit (GPU) in less than one second. The U-Net architecture has also been used in diffusion models for noise removal in images [51]. The basic idea of the U-Net architecture aims to augment a standard shrinking network with successive layers by replacing pooling operations with up-sampling operators. These layers increase the output resolution. Subsequent convolutional layers learn to use this high-resolution information to generate a precise output. A notable innovation of U-Net is the inclusion of several feature channels in the upsampling part. These channels allow the network to transfer context information to higher-resolution layers. Therefore, the expanding path is almost symmetrical with the contracting path, resulting in a u-shaped architecture [47,48,52–54].

**Fig 5:**
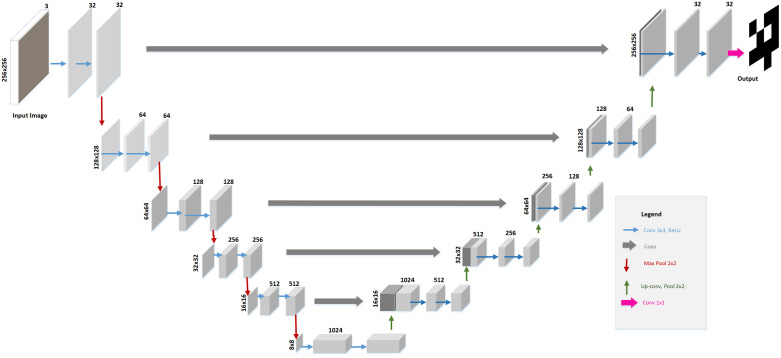
U-Net architecture.

The U-network has a u-shaped architecture with a structure of contracting and expanding paths. The collapsing path is a classical convolutional network consisting of iterative convolution operations, each of which is followed by a corrected linear unit (ReLU) followed by a maximum pooling operation. By combining high-resolution features from the contracting path through a series of up-convolution and merging operations, the expanding path recovers both feature and spatial information [55]. Fig 5 shows the U-Net architecture. As shown in Fig 5, the generated image and block labels are fed into a 6-level U-Net model to predict the classes of blocks on the image. Blue arrows represent 3D (3x3) Convolution and ReLU at the horizontal level, red arrows represent 2x2 MaxPooling between levels, green arrows represent 2x2 Pooling, and pink arrow represents 1x1 Convolution.

## 3. Experimental results

**Fig 6:**
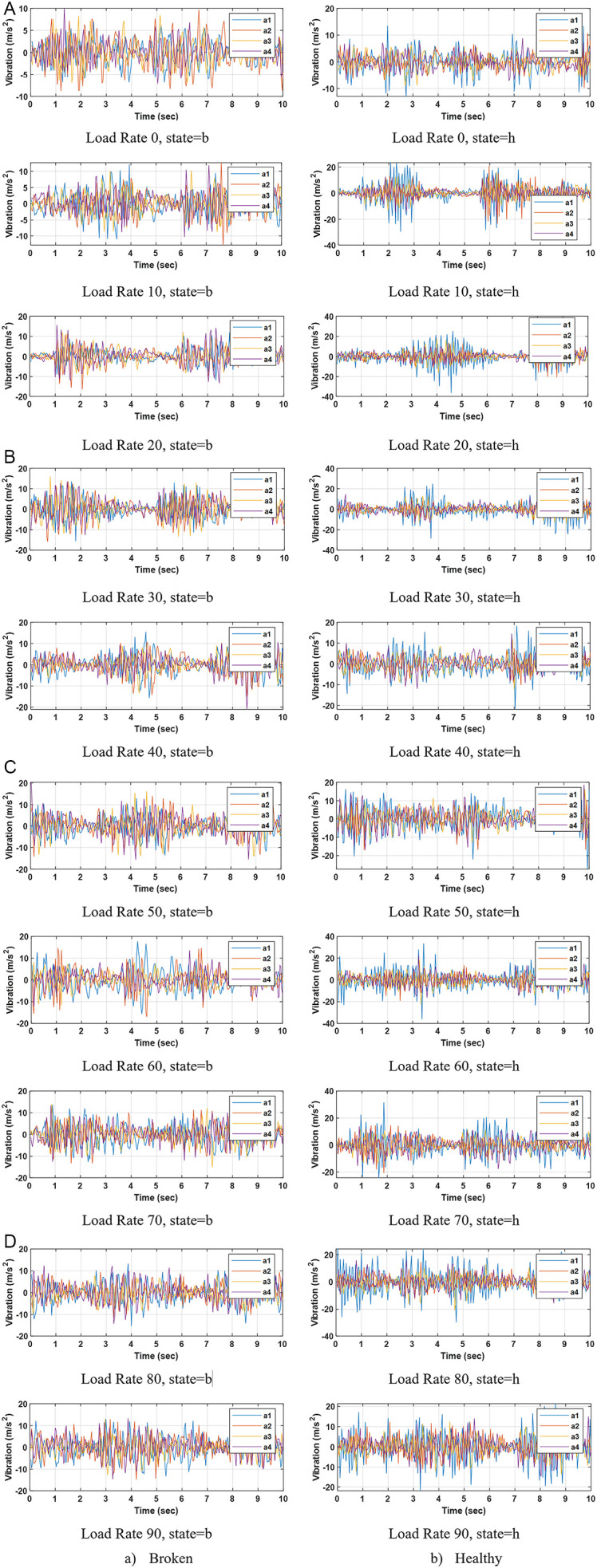
Graphs of broken and healthy gear sensor data at different loads (0%−90%) (a) Broken (b) Heathy.

For the implementation of the proposed method, a computer with Intel i7 8th generation processor and 32-GB memory was used. The application was performed on this computer using MATLAB software. Vibration data graphs for 4 different sensor types at different loads are given in Fig 6. When the graphs for different loads are analyzed, it is seen that the broken gear and healthy gear plots are separated. Therefore, it is seen that high performance results can be obtained. In Fig 6, the horizontal axis shows the 10-second advancement in seconds, and the vertical axis shows the vibration change in m/s². As can be seen, the vibration change plots are clearly separated for different loads.

**Fig 7:**
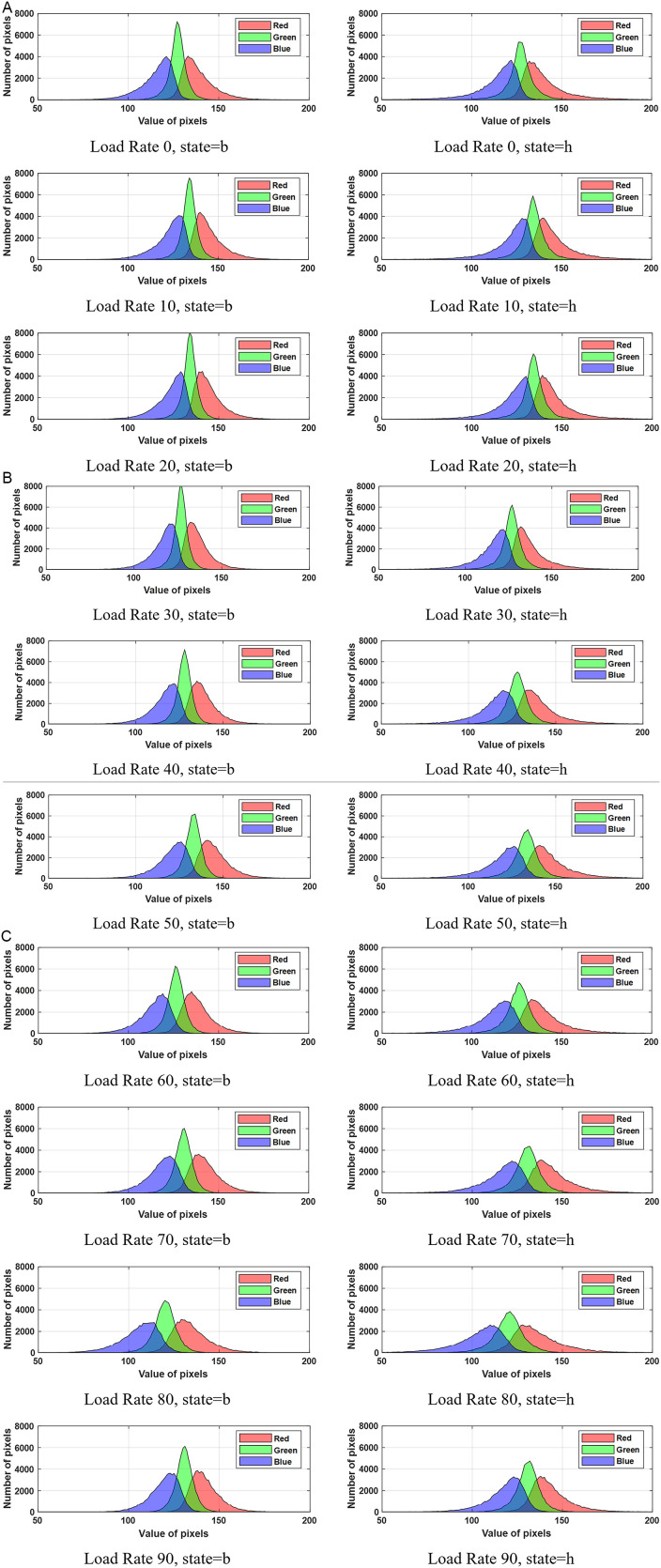
RGB image histogram plots obtained for different loads (a) Broken (b) Healhty.

RGB histogram graphs of the images obtained according to Algorithm 2 are shown in Fig 7. When the plots are analyzed, it can be seen that RGB images for different loads can be segmented with the U-Net model with high performance. In Fig 7, the horizontal axis shows the pixel value variation in the Red, Green and Blue channels in the range [0.255], and the vertical axis shows how many of these pixel values there are for each channel.

**Fig 8:**
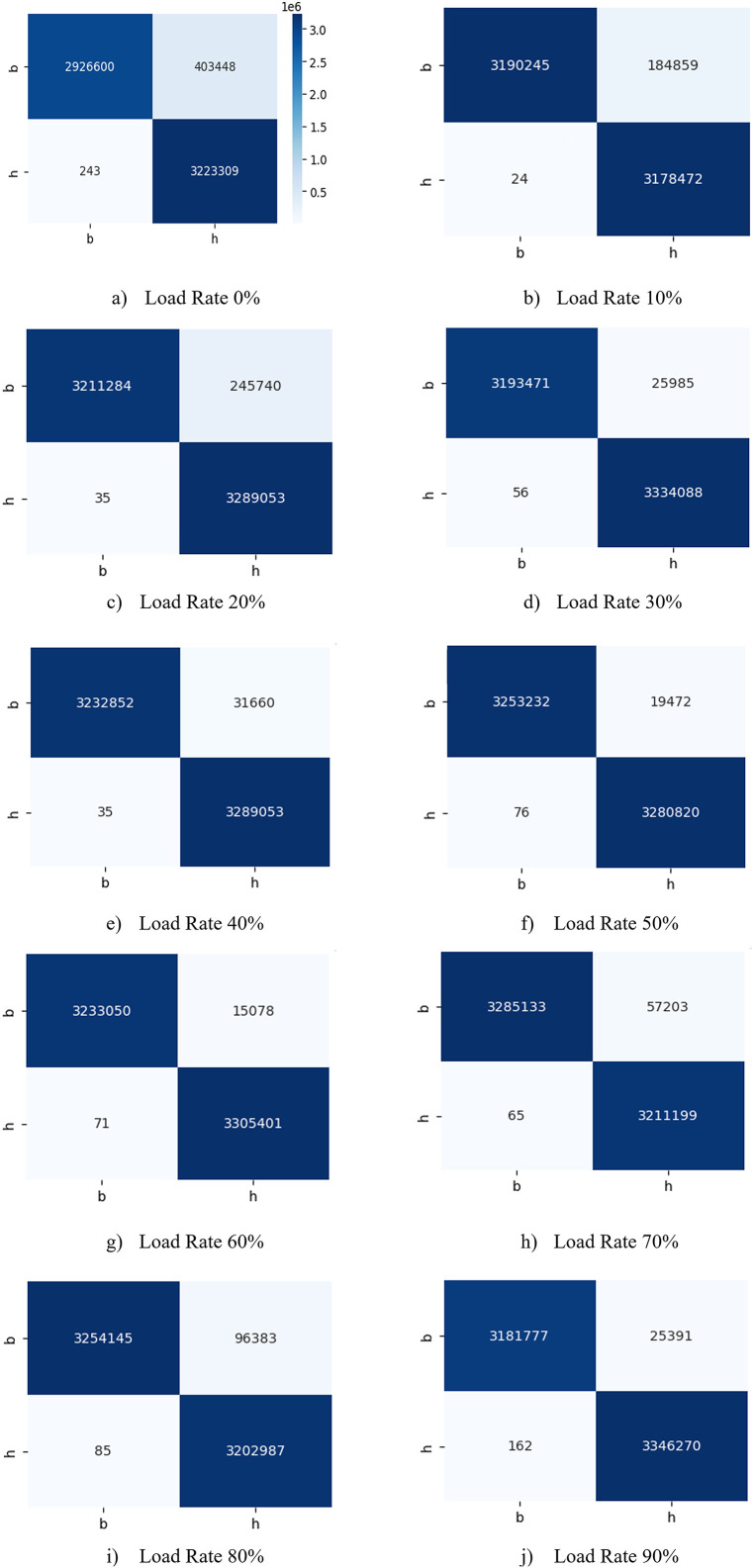
Confusion matrix results for all loads.

Fig 8 shows the confusion matrix results calculated for different loads. The results show that the distribution is generally excellent for all loads. Especially from 30% to 60% and 90% loads the results are more pronounced.

Table 3 shows the results of class-based performance for all loads. A healthy gearbox fault diagnosis accuracy rate of 99.99% is obtained for all loads, as shown in Table 3. For the broken gearbox, 99.53% accuracy was obtained at 60% load.

**Table 3:** Class-based performance results.

Load Rate	Class	Accuracy	IoU	MeanBFScore
**0%**	Broken	87.88	87.87	57.88
	Healthy	99.99	88.86	59.74
**10%**	Broken	94.52	94.52	75.30
	Healthy	99.99	94.50	77.05
**20%**	Broken	92.89	92.89	70.09
	Healthy	99.99	92.64	71.02
**30%**	Broken	**99.19**	99.19	95.75
	Healthy	99.99	99.22	96.42
**40%**	Broken	99.03	99.02	94.49
	Healthy	99.99	99.04	95.17
**50%**	Broken	**99.40**	99.40	96.23
	Healthy	99.99	99.40	96.90
**60%**	Broken	**99.53**	99.53	97.57
	Healthy	99.99	99.54	97.84
**70%**	Broken	98.28	98.28	90.51
	Healthy	99.99	98.24	91.43
**80%**	Broken	97.12	97.12	83.61
	Healthy	99.99	97.07	84.95
**90%**	Broken	**99.20**	99.20	95.78
	Healthy	99.99	99.24	96.25

The general performance results for 2 classes are given in Table 4. When the results are examined, it is seen that they are obtained at 30%, 50%, 60%, and 90% loads, as shown in the confusion matrices in Fig 8. The best result (99.76%) was obtained at 60% load.

**Table 4:** General performance results for different load rates.

Load Rate	GlobalAccuracy	MeanAccuracy	MeanIoU	WeightedIoU	MeanBFScore
**0%**	93.84	93.93	88.37	88.36	58.81
**10%**	97.17	97.26	94.51	94.51	76.17
**20%**	96.25	96.44	92.76	92.77	70.55
**30%**	**99.60**	99.59	99.20	99.20	96.09
**40%**	99.51	99.51	99.03	99.03	94.83
**50%**	**99.70**	99.70	99.40	99.40	96.57
**60%**	**99.76**	99.76	99.53	99.53	97.70
**70%**	99.12	99.14	98.26	98.26	90.97
**80%**	98.52	98.56	97.09	97.09	84.28
**90%**	**99.61**	99.60	99.22	99.22	96.02

The validation accuracy values are presented in Table 5. The obtained validation accuracy results show that the proposed method is consistent.

**Table 5:** Validation accuracy results.

Load Rate	0	10	20	30	40	50	60	70	80	90
**Validation Accuracy**	93.84	97.18	96.25	99.60	99.52	99.70	**99.77**	99.13	98.53	99.61

**Fig 9:**
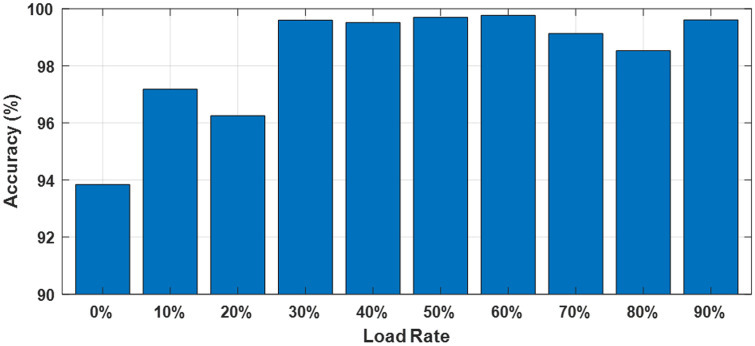
Accuracy bar charts for all loads.

Fig 9 shows the accuracy bar graphs for all loads. In Fig 9, the horizontal axis shows different load ratios, and the vertical axis shows the Accuracy value as a percentage for different loads.

**Fig 10:**
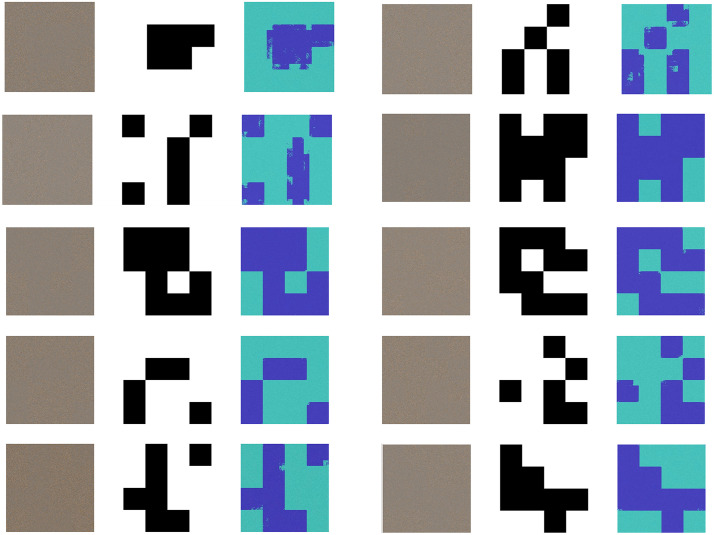
Results obtained as a result of the U-Net implementation (a) Image (b) Ground Truth (c) U-Net (d) Performance Metrics (%).

Fig 10 shows the results obtained with U-Net on sample images and labels.

## 4. Discussion

**Fig 11:**
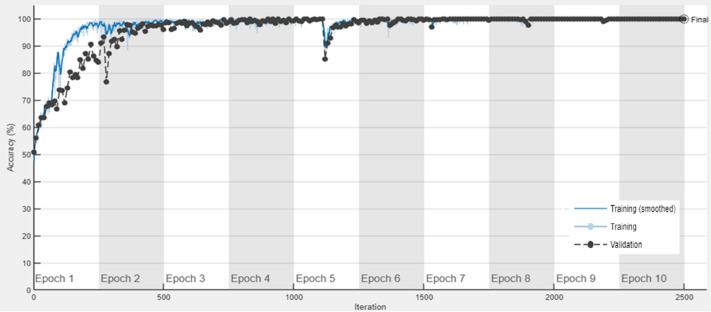
Accuracy graphs (5000 images, 10 epochs).

**Fig 12:**
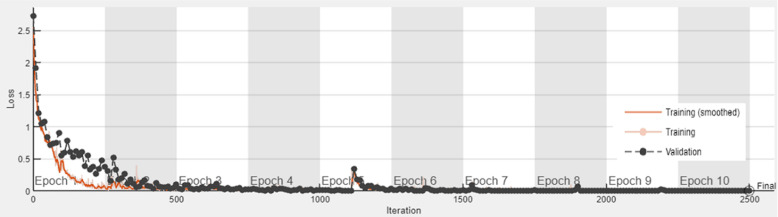
Loss plots (5000 images, 10 epochs).

For each load, 500 images were created, and the overall results were obtained by applying U-Net to 5000 images. Figs 11 and 12 show the accuracy and loss graphs for the training and validation data for 10 epochs. In Fig 11, the horizontal axis shows the change in iteration for 10 epochs, and the vertical axis shows the change in Accuracy relative to the change in iteration, as a percentage. Similarly, in Fig 12, the horizontal axis shows the iteration change for 10 epochs, and the vertical axis shows the Loss change relative to the iteration change, as a percentage. As can be seen, the changes in Accuracy and Loss have stabilized since Epoch 6. Therefore, 10 epochs are sufficient for the training process.

**Fig 13:**
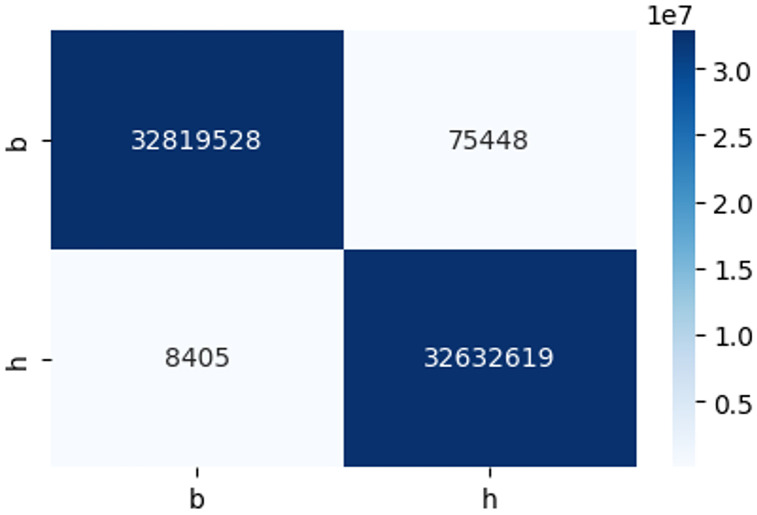
Confusion matrix for 5000 images training.

Fig 13 shows the confusion matrix obtained as a result of training.

The accuracy, precision, recall, F1-score, and IoU (mAP) values, which are the performance metrics given in Table 6, were calculated according to Equations (5), (6), (7), (8), and (9), respectively. In other words, the area overlapping the common area must overlap one-to-one. This is a state-of-the-art approach that pushes the limits. Accuracy (Equation 5) represents the percentage of correct predictions made by the model; Precision (Equation 6) indicates the proportion of samples predicted as broken that are actually faulty; Recall (Equation 7) measures the proportion of truly faulty samples that are correctly identified by the model; F1-score (Equation 8) summarizes the overall performance as the harmonic mean of Precision and Recall; and IoU (mAP) (Equation 9) represents the average overlap ratio between the predicted and ground truth segmentation masks across all images.

**Table 6:** Comparison with other methods for gearbox diagnostics.

References	Method(s)	IoU (mAP) (%)	Accuracy (%)	Precision (%)	Recall (%)	F1-Score (%)
Ahmed, I. et al [56], 2023	Deep Learning, Autoencoder		91.00	98.00	83.00	–
NA Raji et al [10], 2024	Machine Learning – AdaBoost Classifier ET	–	87.56	88.36	86.38	87.36
Sohaib Arshad Mayo et al [57], 2024	Deep Learning – Keras Sequential API	–	98.63	99.20	92.30	96.24
Proposed Method	Deep Learning – U-Net Segmentation Model	**99.74**	**99.87**	**99.77**	**99.97**	**99.87**


$$\begin{array}{c} Accuracy=\frac{TP+TN}{TP+TN+FP+FN} \end{array}$$ (5)

$$\begin{array}{c} Precision=\frac{TP}{TP+FP} \end{array}$$ (6)

$$\begin{array}{c} Recall=\frac{TP}{TP+FN} \end{array}$$ (7)

$$\begin{array}{c} F1\text{-}Score=\frac{2TP}{2TP+FP+FN} \end{array}$$ (8)

$$\begin{array}{c} IoU(mAP)=\frac{\text{1}}{totalimages}\sum_{i=\text{0}}^{totalimages}{Precision}_{i}{Recall}_{i} \end{array}$$ (9)

**Fig 14:**
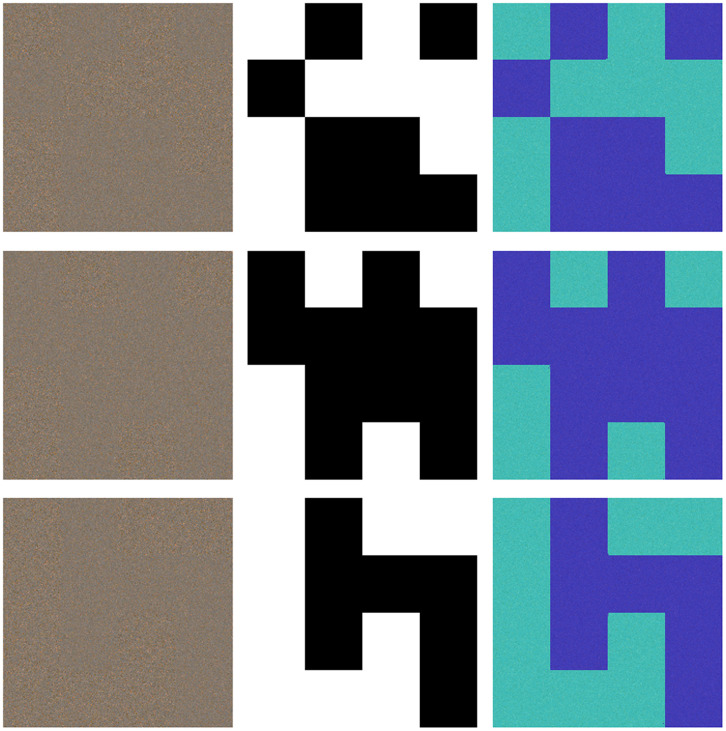
Total of 5000 images and U-Net overlapping images (a) Image (b) Ground Truth (c) U-Net.

Fig 14 shows the segmentation sample images obtained from U-Net for 5000 images.

Table 6 shows that recent studies have focused on deep learning methods. In NA Raji et al. [10], it is seen that similar machine learning methods do not exceed 87%−88% accuracy values. In Ahmed, I. et al. [56], a 91% accuracy value was obtained using a deep learning-based autoencoder method. In Sohaib Arshad Mayo et al. obtained a 98.68% accuracy result close to the state-of-the-art using the pre-trained Keras Sequential API deep learning model. The proposed method yielded state-of-the-art results with 99.87% accuracy.

The segmentation performed in this study does not aim to localize faults on the physical components of the gear system. Instead, it represents patterns in an artificial image space constructed from statistical features extracted from vibration data. Therefore, the resulting segmentation output highlights feature regions associated with faults, rather than indicating physical locations.

The proposed method does not aim at physical fault localization but rather at global classification through a spatially structured representation. In this study, segmentation is used as a representation learning tool to capture inter-sensor relationships and local patterns. However, the outputs represent patterns in the feature space rather than physical locations. Furthermore, the computational cost of the model requires optimization for real-time applications.

The high accuracy and IoU values obtained demonstrate that the proposed method is highly successful in distinguishing between healthy and faulty states; however, it should be noted that this performance is also influenced by the binary nature of the problem. The main contribution of the proposed approach is the integration of multi-sensor data through an image-based representation of vibration signals, enabling the learning of local patterns. In this context, segmentation does not aim at physical localization but is instead used as a representation mechanism to capture patterns in the feature space. Compared to existing methods in the literature, the proposed approach offers not only high performance but also an alternative data representation strategy. However, the computational cost of the model requires optimization for real-time applications.

## 5. Conclusion

While gearbox fault diagnosis is performed with approximately 90% accuracy using classical machine learning methods, deep learning methods have been used with high accuracy. In this study, an image-based convolutional approach is adopted instead of the classical deep learning approaches previously used with numerical data. For this purpose, these data were converted into images, and a state-of-the-art result (99.87%) was obtained using the pre-trained U-Net deep learning model for image segmentation. In addition, the mAP value for IoU = 99.74 gave a state-of-the-art result of 99.74%. This proved the effectiveness of the proposed approach.

Rather than directly proposing a new classification algorithm, this study presents an image-based representation of vibration data and a segmentation-based learning approach built upon this representation. In this respect, the contribution should be regarded not only as a performance improvement but also as a methodological alternative for data representation. In future work, we plan to apply our proposed approach to similar diagnostic problems. A portable application using this approach can be developed in the industry, and an error-free diagnosis can be made.

### 5.1. Limitations

While the proposed method demonstrates high accuracy and state-of-the-art performance, several limitations should be considered. First, the study is formulated as a binary classification problem (healthy vs. faulty). Although this design enables controlled evaluation under varying load conditions, it does not fully capture the complexity of real-world transmission diagnostics, where multiple failure types (e.g., wear, misalignment, and lubrication defects) may coexist. Therefore, the proposed method should be regarded as a preliminary yet efficient validation framework rather than a comprehensive fault diagnosis system. Second, the proposed approach relies on a synthetic image generation methodology based on statistical features (maximum, mean, and minimum). While this transformation facilitates the use of image-based deep learning models, it may result in the loss of important information related to temporal dependencies and frequency-domain characteristics, which are often critical in vibration analysis.

Third, segmentation masks are generated using a block-based labeling strategy, in which a single class is assigned to each block. Although this simplifies the learning process, it does not accurately reflect pixel-level fault localization observed in real-world scenarios. Consequently, the segmentation task is artificially constructed, which may limit its interpretability and practical applicability.

Fourth, the study does not include a direct comparison with established 1D approaches, such as 1D CNNs, LSTM networks, or time–frequency representation (TFR)-based methods [58]. Since these techniques are widely used in vibration-based fault diagnosis and often achieve high accuracy, their absence limits the ability to fully position the proposed method within the existing literature. Finally, although the dataset includes multiple load conditions, all data were collected using a controlled experimental setup (SpectraQuest Transmission Simulator). This may restrict the generalizability of the results to real industrial environments, where noise, sensor placement variability, and operational uncertainties are more prominent.

### 5.2. Future work

Future studies will address these limitations and extend the proposed framework in several directions. First, the method will be evaluated in multi-class and multi-failure diagnostic scenarios. By extending the segmentation masks to include multiple fault categories, the proposed methodology can better represent realistic industrial conditions in which different fault types may occur simultaneously. Second, more advanced signal representations will be explored. In particular, integrating time–frequency representations (e.g., spectrograms and wavelet transforms), or combining them with the proposed RGB encoding, may improve feature preservation and enhance model performance. Third, the image generation process will be refined to reduce information loss and better capture temporal dynamics. This may involve sliding window techniques, adaptive feature extraction methods, or hybrid representations that combine statistical and ordinal features.

Fourth, a comprehensive benchmarking study will be conducted against advanced 1D and 2D deep learning models, including 1D CNNs, LSTMs, and TFR-based CNN architectures. This will provide a clearer understanding of the strengths and limitations of the proposed approach. Fifth, the proposed method will be validated using real-world industrial datasets to evaluate its robustness and generalization capability under practical conditions. Finally, future work will focus on developing lightweight and real-time implementations of the model for deployment in embedded or edge-based condition monitoring systems.
